# Evolution of major milk proteins in *Mus musculus *and *Mus spretus *mouse species: a genoproteomic analysis

**DOI:** 10.1186/1471-2164-12-80

**Published:** 2011-01-28

**Authors:** Nisrine Boumahrou, Claudia Bevilacqua, Christian Beauvallet, Guy Miranda, Sanda Andrei, Emmanuelle Rebours, Jean-Jacques Panthier, Sylvain Bellier, Patrice Martin

**Affiliations:** 1 INRA, UR1313 Génétique animale et Biologie intégrative UMR 1313, Equipe LGS, F-78350 Jouy-en-Josas, France; 2 Universitatea de Stiinte Agricole si Medicina Veterinara Cluj-Napoca, Romania; 3 Institut Pasteur, Génétique Fonctionnelle de la Souris; CNRS URA2578, F-75724 Paris, France; 4 INRA, UMR955 Génétique Fonctionnelle et Médicale, F-94704 Maisons-Alfort, France

## Abstract

**Background:**

Due to their high level of genotypic and phenotypic variability, *Mus spretus *strains were introduced in laboratories to investigate the genetic determinism of complex phenotypes including quantitative trait loci. *Mus spretus *diverged from *Mus musculus *around 2.5 million years ago and exhibits on average a single nucleotide polymorphism (SNP) in every 100 base pairs when compared with any of the classical laboratory strains. A genoproteomic approach was used to assess polymorphism of the major milk proteins between SEG/Pas and C57BL/6J, two inbred strains of mice representative of *Mus spretus *and *Mus musculus *species, respectively.

**Results:**

The milk protein concentration was dramatically reduced in the SEG/Pas strain by comparison with the C57BL/6J strain (34 ± 9 g/L *vs*. 125 ± 12 g/L, respectively). Nine major proteins were identified in both milks using RP-HPLC, bi-dimensional electrophoresis and MALDI-Tof mass spectrometry. Two caseins (β and α_s1_) and the whey acidic protein (WAP), showed distinct chromatographic and electrophoresis behaviours. These differences were partly explained by the occurrence of amino acid substitutions and splicing variants revealed by cDNA sequencing. A total of 34 SNPs were identified in the coding and 3'untranslated regions of the SEG/Pas *Csn1s1 *(11), *Csn2 *(7) and *Wap *(8) genes. In addition, a 3 nucleotide deletion leading to the loss of a serine residue at position 93 was found in the SEG/Pas *Wap *gene.

**Conclusion:**

SNP frequencies found in three milk protein-encoding genes between *Mus spretus *and *Mus musculus *is twice the values previously reported at the whole genome level. However, the protein structure and post-translational modifications seem not to be affected by SNPs characterized in our study. Splicing mechanisms (cryptic splice site usage, exon skipping, error-prone junction sequence), already identified in casein genes from other species, likely explain the existence of multiple α_s1_-casein isoforms both in SEG/Pas and C57BL/6J strains. Finally, we propose a possible mechanism by which the hallmark tandem duplication of a 18-nt exon (14 copies) may have occurred in the mouse genome.

## Background

Classical laboratory inbred strains of mice offer the most valuable model system for medical research, allowing for instance the analysis of complex traits. However, laboratory strains were derived from a limited number of founding mice that belonged to several *Mus musculus *subspecies: *Mus m. domesticus, Mus m. musculus, M. m. castaneus *and the hybrid *M. m. molossinus *[1]. Therefore, their genetic variation does not encompass the diversity seen in mice trapped in many geographical areas in the wild. To overcome this lack of polymorphism, strains that belong to different species of the *Mus *genus have recently been established from wild progenitors. Among these emerges the short-tailed species *Mus spretus*, a western Mediterranean mouse, that splitted from *Mus musculus *around 2.5 million years ago [1]. Although they are sympatric, these species rarely generate hybrids in nature. Under laboratory conditions, they produce viable and fertile offspring with a large number of polymorphisms of natural origin, but male offspring are sterile. Strains of *Mus spretus *are frequently utilized in combination with *Mus musculus *strains for quantitative trait loci (QTL) studies, due to their high degree of sequence and phenotypic diversity [2]. Indeed, *Mus spretus *mice have been valuable for the identification of loci contributing to differences in immune response [3] and were used to generate the first high-density genetic map for the mouse [4].

Any inbred strain derived from *Mus spretus *exhibits on average a SNP (single nucleotide polymorphism) in every 100 base pairs when compared with any of the classical laboratory strain. By comparison, the frequency of SNPs between humans is roughly one order of magnitude lower. However, a comparison study between the brain proteomes of *Mus musculus *and *Mus spretus *[5] revealed a considerable discrepancy between the frequency of qualitative protein polymorphisms detected by 2-DE (around 8%) and the frequency predicted on the basis of SNPs (possibly up to 90%). The protein polymorphism between mouse species has been analyzed from different tissues [5,6]. Milk protein polymorphism plays an important role in genetic diversity analysis and phylogenetic studies. In addition, it contributes to improve our understanding of mammary evolution and of the role and sustainable use of genetic variation in farm animals. To our knowledge, although mice can be a pertinent model system for the identification of candidate genes for QTL of milk production traits in cattle [7,8], no proteomic studies have been carried out so far to analyse the diversity of mouse milk proteins and their polymorphism across *Mus *species.

In this report, we compared the major milk proteins between SEG/Pas and C57BL/6J strains. SEG/Pas was derived from *Mus spretus, *while C57BL/6J is a reference laboratory strain belonging to the *Mus musculus *species. The majority of the genome sequence data available for *Mus spretus *are from SPRET/Ei, while the SEG/Pas genome sequence data are limited and even absent concerning the major milk protein encoding genes.

In our study, polymorphisms occurring in major milk proteins between C57BL/6J and SEG/Pas were analyzed and characterized. We used both proteomic and genomic approaches to highlight differences between both strains. Interestingly, three of the main milk proteins showed structural differences, with allelic variation and some flexibility in splicing patterns of primary transcripts arising from the corresponding genes.

## Methods

### Mice

Mice from the C57BL/6J and SEG/Pas inbred strains were housed at the INRA (Jouy-en-Josas, France) and Institut Pasteur (Paris, France) research centers, respectively. Milk samples were collected during the first part of lactation, i.e. at day (d) 4, 8 and 10 from parturition, and the second part of lactation (d14). Females were separated 2 hours before milking, injected with 0.2 U synthetic oxytocin (CEVA SANTE ANIMALE, Libourne, France) and then anesthetized by intraperitoneal injection (0.01 mL/g of body weight) of a solution containing 1 mL Imalgène 1000 (MERIAL, Lyon, France), 0.5 mL Rompun (Bayer Pharma, Puteaux, France) in a final volume of 10 mL water. After milking, the collected milk samples were diluted with water in a 1:3 (v:v) ratio, skimmed by centrifugation (4,000 rpm; 4°C, 15 min) and stored at -80°C. Animal care and experimentations were in accordance with guidelines of the International Guiding Principles for Biomedical Research.

### RP-HPLC analysis

Protein concentration of milks was determined using a Bradford assay [9]. Clarified skimmed milk samples were analysed by RP-HPLC as previously described [10]. Chromatographic peaks were collected and dried in a vacuum system.

### SDS-Poly Acrylamide Gel Electrophoresis (SDS-PAGE) analysis

Pooled chromatographic fractions corresponding to each peak were subsequently separated by SDS-PAGE as previously described [10]. Proteins (25 μg) from skimmed milk collected on day 4, 8, and 14 of lactation were also analyzed by 12.5% SDS-PAGE mini-gels.

### 2D-Gel Electrophoresis analysis

Milks were further characterized by 2D Electrophoresis. Skimmed milk samples containing 350 μg of proteins were diluted in a rehydration solution containing urea/thiourea (7M/2M), chaps (4%), DTT 100 mM, IPG pH 4-7 carrier ampholytes (0.5%) and a trace of bromophenol blue to obtain a final volume of 450 μL. The rehydration step was performed in active mode (50 volts) on 24-cm Immobiline drystrip pH 4-7 for 12 hours in a Protean IEF Cell (Bio-Rad). Afterwards, isoelectric focusing (IEF) was run by increasing progressively the voltage to reach a plateau at 10 kV and a total applied voltage of 74 kVh in 13 hours. The focused proteins were successively reduced (10 mg/ml DTT), and alkylated (200 mM iodoacetamide) and the strips equilibrated in a solution of 6 M urea, 2% SDS, 0.375 M Tris pH 8.8 and 30% glycerol. The strips were then loaded on the top of home-cast linear 12.5% polyacrylamide gels (25 × 20 cm) and were embedded in a solution of 1% of agarose. SDS-PAGE were performed in an Ettan Dalt six electrophoresis unit (GE Healthcare) at 5 mA/gel for 30 min, 8 mA/gel for 30 min and finally at 1.5 W/gel until the front of migration reached the bottom of the gels. Proteins were stained with BioSafe Coomassie blue (Bio-Rad) and the gels were imaged on a LabImager (GE Healthcare).

### MALDI-TOF Mass Spectrometry

Protein bands or spots from 1-D or 2-D gels were excised and the proteins were in-gel digested with trypsin. Tryptic peptides were analysed on a Voyager DE-STR Maldi-Tof mass spectrometer (Applied Biosystems Inc., Framigham, MD). Peptides co-crystallized with α-CHCA matrix (5 mg/mL dissolved in Acetonitrile/TFA 50/0.3%), were desorbed and ionized with a nitrogen laser at 337 nm in the positive ion mode and delayed extraction. Spectra were internally calibrated using both trypsin autolysis peaks (842.5090 Da and 2211.1040 Da) in Data Explorer software (Applied Biosystems). After deisotoping, the peptide mass lists were searched against the SwissProt 2007 and UniProt 2007 databases with the MS-Fit Protein Prospector software (http://www.expasy.org).

### LTQ-Orbitrap Mass Spectrometry

HPLC fractions corresponding to the Whey Acidic Protein (WAP) were solubilized in an acetonitrile/water/formic acid 50/49/1 (v/v/v) solution. The sample solution was infused at a flow rate of 100 μL/min in an electrospray source of a LTQ-Orbitrap XL (Thermo Fisher Scientific, USA, MA). Proteins were ionized with a spray voltage of 1 kV and a temperature of 200 °C. Spectra were averaged for 5 min and deconvoluted with MaxEnt algorithm.

### RNA extraction and RT-PCR

Mammary glands from milked animals killed by cervical dislocation were dissected. The mammary tissue was collected from C57BL/6J and SEG/Pas lactating mice. Total RNA was extracted from the mammary tissue samples using RNAlater (Invitrogen Life Technologies, Carlsbad, California, USA) according to the manufacturer's instructions. RNA quantity, quality and purity were analyzed as previously described [11]. RNA were mixed with 1 μL of 2/3 random primers (3 μg/μL) and 1/3 oligo (dT)_20 _(50 μM) to a final volume of 20 μL. Synthesis of the first complementary DNA strand was performed with reverse transcriptase (RT) (SuperScript™ III, Invitrogen) according to the manufacturer's guidelines. Briefly, cDNA synthesis was carried out at 50°C for 45 min. RT was inactivated at 70°C for 15 min. In order to remove RNA complementary to the first cDNA strand, 2 units of *E. coli *RNaseH were added. Finally, the mix was incubated at 37°C for 20 min.

### PCR amplification of cDNA, subcloning and sequencing

Primers were designed to amplify the cDNA from *Csn1s1*, *Csn2 *and *Wap *transcripts (Table 1). The primers were purchased from MWG Biotech (France). PCR products were analyzed by electrophoresis in the presence of ethidium bromide (0.1 μL/mL) in agarose gel in tris/borate/EDTA buffer. The PCR products for *Wap *and *Csn2 *were directly sequenced using primer pairs used for PCR. The PCR products yielded for *Csn1s1 *were cloned in pCR^®^4-TOPO^® ^plasmid vector (Invitrogen) and transformed into TOP10 *E. coli *cells according to the protocol supplied in the Kit for sequencing (Ref. K4575-J10). Clones were tested by PCR, using M13 reverse and forward primers, and recombinant plasmids were sequenced from both strands by GATC Biotech (Konstanz, Germany).

**Table 1:** Primers used to amplify the cDNA target of the *Csn1s1*, *Csn2 *and *Wap *genes.

Genes	Primers	Primer sequence 5' → 3'
***Csn1s1***	Forward Reverse	ATG AAA CTC CTC ATC CTC ACC TG CAA TCT CAG TTA CTA CAC ACA ATT

***Csn2***	Forward Reverse	ATG AAG GTC TTC ATC CTC GC TCA ACT CCA TAT TGA ACA CTT AT

***Wap***	Forward Reverse	GTT GCC TCA TCA GCC TTG TT TTA GCA GCA GAT TGA AAG CAT

## Results

### Analysis of the milk protein fraction at mid-lactation

**Figure 1:**
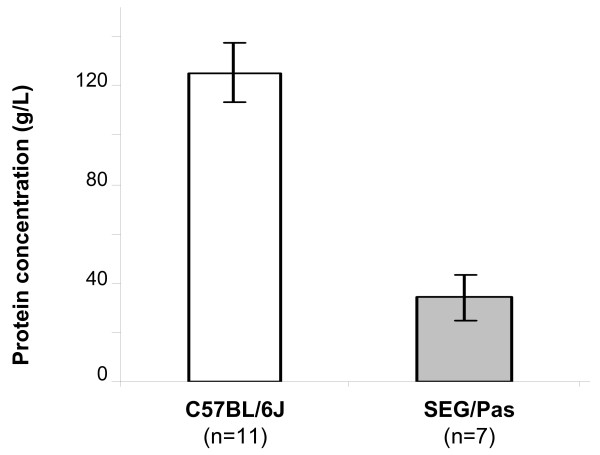
**Mean protein concentration of skimmed milks from C57BL/6J and SEG/Pas mice, at 10 days of lactation**. The number of mice used in each population is given in parentheses. Error bars correspond to the standard error.

The protein concentration of milks collected on the 10^th ^day of lactation from SEG/Pas and C57BL/6J mice differed significantly : 34 ± 9 g/L *vs*. 125 ± 12 g/L, respectively (Figure 1), being almost four times lower for SEG/Pas than for C57BL/6J.

**Figure 2:**
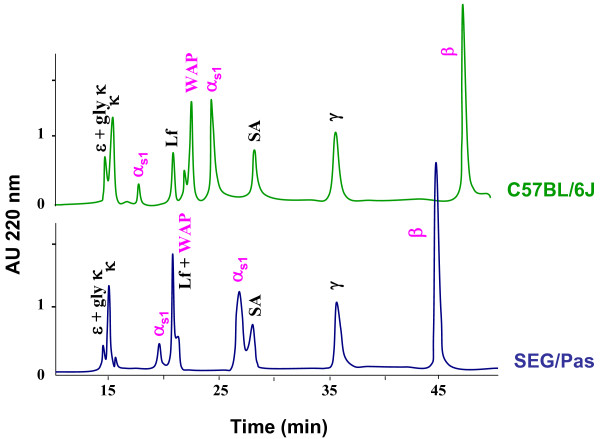
**RP-HPLC elution profiles of C57BL/6J (green) and SEG/Pas (blue) skimmed milks samples at 10 days of lactation**. Clarified milk samples (100 μl; ca. 350 μg of proteins) were injected on a Jupiter C5 Reverse Phase column (300 Å pore size, 5 μm, 150 × 4.6 mm; Phenomenex, Paris, France). The elution was achieved by a two steps linear gradient: 100% A (TFA/H2O 1.1:1000 v/v) to 20% solvent B (TFA/CAN 1:1000 v/v) over 10 min followed by 37% solvent B to 60% over 45 min at a flow rate of 1ml/min. The column was kept at 40°C. Proteins showing a different behavior between mouse species are indicated in pink.

The RP-HPLC method previously described [10] allowing protein polymorphism detection in *Mus musculus *strains was applied to compare the major protein fraction of SEG/Pas and C57BL/6J milks. Elution profiles of the main milk proteins thus obtained are given in Figure 2. The protein content of each peak was subsequently analyzed by SDS-PAGE and protein bands extracted from the gel were identified through Peptide-Mass Finger printing (PMF), using a MALDI-TOF mass spectrometer. Depending on the mouse strain, β- and α_s1_-caseins as well as WAP exhibited different retention times.

To assess that the chromatographic retention time differences are real and not due to experimental artifact, milk samples were first analyzed in duplicate. Identical elution patterns were recurrently obtained with the same sample and with several individual milks, at the same stage of lactation. β-casein and WAP from SEG/Pas were eluted earlier than those from C57BL/6J. Two isoforms of α_s1_-casein were observed, as previously described [10]. However, they showed a longer retention time in SEG/Pas than those from C57BL/6J.

**Figure 3:**
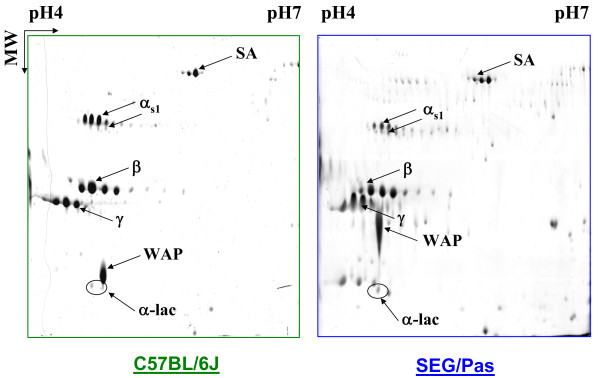
**2-DE of C57BL/6J and SEG/Pas skimmed milks at 10 days of lactation**. The protein spots showed by arrows were identified by a PMF analysis as being Serum Albumin (SA), α_s1_-casein (α_s1_), β-casein (β), γ-casein (γ), Whey Acidic Protein (WAP) and α-lactalbumin (α-lac).

To further confirm and characterize such a protein polymorphism, milk samples from C57BL/6J and SEG/Pas were subsequently analysed by 2-DE (Figure 3). The main spots were identified by MALDI-TOF mass spectrometry. α_s1_-, β- and γ-caseins, as well as α-lactalbumin and serum albumin displayed several spots in the gels. Conversely, WAP was present as a single spot. The major milk proteins were all identified, except κ-casein, probably because this glycosylated protein has a pI below 4.0 which was the lower limit of the IEF strip used here. In SEG/Pas, WAP showed a different electrophoretic mobility, with a lower apparent pI and a higher molecular mass, compared to C57BL/6J. Relative proportions of α_s1_-casein isoforms were also species-dependent. In 2D gels, the minor α_s1_-casein isoforms appeared to be in higher amount in SEG/Pas, whereas the major α_s1_-casein isoform was less abundant compared to C57BL/6J. This result was in accordance with the chromatographic pattern shown in Figure 2. Finally, 2-DE analysis allowed highlighting α-lactalbumin, which was not detected in our chromatographic analysis conditions.

### Evolution of major milk proteins over the lactation period

**Figure 4:**
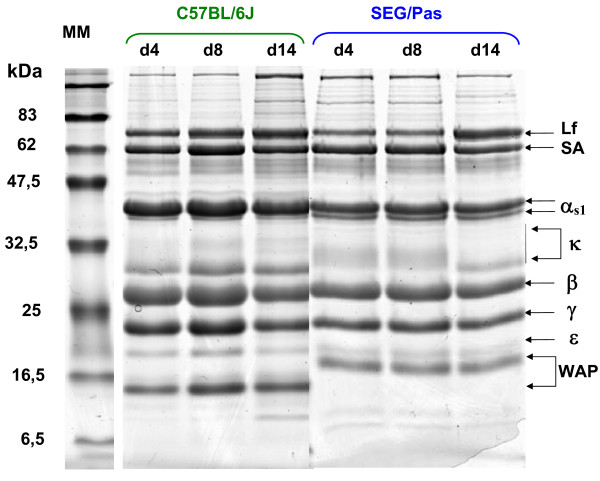
**SDS-PAGE analysis of skimmed milks collected from C57BL/6J and SEG/Pas mice at 4, 8, and 14 days of lactation**. Names of the main proteins are indicated (Lf: Lactoferrin, SA: Serum Albumin, α_s1_: α_s1_-casein, β: β-casein, γ: γ-casein, ε: ε-casein, WAP: Whey Acidic Protein). MM: Molecular markers.

To test whether the overall major protein composition varies over the lactation period in milk from SEG/Pas and C57BL/6J, milk was collected from females at day (d) 4, 8, and 14 after parturition. Skimmed milks were analysed in SDS-PAGE. Mono dimensional electrophoresis patterns of proteins (Figure 4) at d4, d8 and d14 displayed no visual differences within strains, except for κ-casein. In the SEG/Pas strain, at day 4, κ-casein appeared as a diffused band, between 30 and 37 kDa. This κ-casein form declined progressively, whereas a sharper band (30 kDa) became predominant during the second part of lactation (d14). By contrast, as far as C57BL/6J milk is concerned, κ-casein appeared as a more resolved and intense band at *ca. *30 kDa, whatever the stage of lactation and topped by a smear of which the intensity increases with the lactation stage.

### Csn1s1 polymorphism

**Figure 5:**
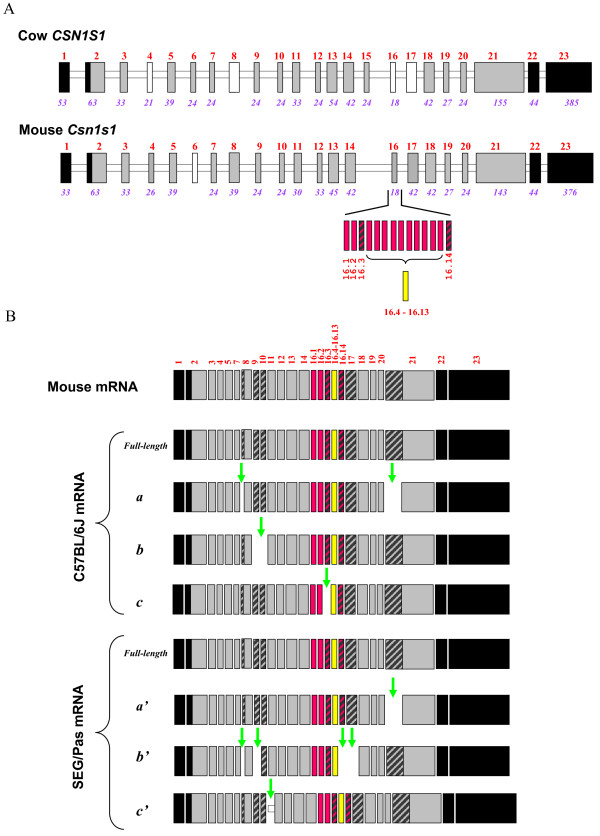
**Schematic representation of the intron/exon structure organization of cow *CSN1S1 *and mouse *Csn1s1 *genes (panel A) and of the corresponding matured transcripts (panel B) found in the mammary tissue of C57BL/6J and SEG/Pas mouse strains**. Open bars represent introns; exons are depicted by large black (3'and 5'untranslated regions) or grey (exons encoding preproteins) boxes, hatched blocks indicate potentially skipped sequence, pink and yellow blocks refer to repeats of exon 16 and no-fill blocks represent exons that are constitutively absent from mRNA according to [29,37]. Numbers above blocks indicate exon number; numbers below (italics) indicate exon size (in bp). Vertical green arrows indicate exons (or sequences) skipped (or included) during the course of the splicing process. C57BL/6J: *a*, deletion of the first codon of exon 8 and of the first 57 nucleotides of exon 21, *b*: exon-skipping of exon 9 and 10, *c*: exon-skipping of exon 16.3. SEG/Pas: *a', *deletion of the first 57 nucleotides of exon 21; *b', *deletion of the first codon of exon 8 and exon-skipping of exons 9, 16.14 and 17; *c'*, insertion of the last 33 nucleotides of intron 10.

To characterize *Csn1s1 *polymorphism, mRNAs encoding α_s1_-casein were isolated from SEG/Pas and C57BL/6J mammary glands, reverse transcribed, amplified, and cloned. Clones showing inserts of different sizes were selected and sequenced. Four *Csn1s1 *cDNAs were found in each species. Figure 5 shows the transcripts identified in SEG/Pas and C57BL/6J tissues together with the intron/exon structure organization of the mouse *Csn1s1 *gene. Full-length nucleotide sequences of the SEG/Pas and C57BL/6J *Csn1s1 *mRNA were determined (GenBank:GU983862 and GenBank:NM_007784.2, respectively). Alignment of SEG/Pas and C57BL/6J *Csn1s1 *cDNA sequences showed 5 single nucleotide polymorphisms (SNPs) in the 3'UTR and 11 SNPs in the coding region; five of them correspond to amino acid substitutions (V53I, E124K, L135F, Y231H, K293E) in the SEG/Pas strain. Three internally deleted transcripts were identified from C57BL/6J mammary gland. They corresponded to: (*a*) cryptic splice site usages occurring within exon 8 (for comparative purposes we adopted a generic exon numbering, valid for all species for which the CSN1S1 casein gene sequence is known and that we propose to use then), leading to the deletion of its first codon (CAG), and within exon 21 of which 57 nucleotides are lost during the course of the primary transcript processing; (*b*): skipping of exons 9 and 10; (*c*) skipping of exon 16.3. Likewise, two internally deleted transcripts were also identified in SEG/Pas mammary gland, due to the same cryptic splice site usages, leading to (*a'*) the loss of 57 nucleotides in exon 21; (*b'*) deletion of the first codon (CAG) in exon 8 and exon-skipping events affecting exons 9, 16.14 and 17. An additional transcript, longer than the proper mRNA, was also identified. This isoform results from insertion of 33 nucleotides from the 3' end of intron 10. None of these deletions or insertion shifted the reading frame, but the putative proteins encoded by these differentially spliced transcripts differ by their molecular weights and potential phosphorylation sites (Table 2).

**Table 2:** α_s1_-casein from C57BL/6J and SEG/Pas mouse strains.

	**C57BL/6J α**_**s1**_**-casein**				**SEG/Pas α**_**s1**_**-casein**			
	
	Full-length	*a*	*b*	*c*	Full-length	*a'*	*b'*	*c'*
**AA**	298	278	282	292	298	279	269	309
**MW (Da)**	34,042	31,564	32,421	33,390	34,064	31,707	30,796	35,182
**pI**	5,70	5,88	6,31	5,70	5,80	5,98	5,27	5,80
**PP**	11	11	10	11	11	11	11	10

Comparison of the main structural features of the mouse α_s1_-casein isoforms deduced from the transcripts sequences found in C57BL/6J and SEG/Pas mouse strains. Number of amino acids (AA), molecular weight (MW), isoelectric point (pI), and number of potential phosphorylation sites (PP) of the different mouse α_s1_-casein isoforms found in the C57BL/6J and SEG/Pas mouse strains. The α_s1_-casein isoforms *a*, *b*, *c*, *a'*, *b'*, *c' *refer to the sequences of the corresponding matured transcripts (Figure 5).

Altogether, these data reveal a "loud" polymorphism in the *Csn1s1 *transcripts in both C57BL/6J and SEG/Pas strains, giving rise to a large diversity of expression products.

### Csn2 polymorphism

**Figure 6:**
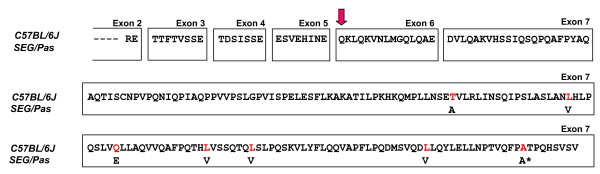
**Alignment of the amino acid sequence of β-casein as deduced from the cDNA obtained from C57BL/6J and SEG/Pas**. Peptides sequences are split into blocks of amino acid residues to visualize the exonic modular structure of the protein as deduced from known splice junctions of the *Mus musculus *sequence. Red amino acids in C57BL/6J sequence indicate the amino acid substitutions in SEG/Pas sequence. A* depicts a conservative mutation in the corresponding codon (GCC-> GCT in *M. spretus*). The pink vertical arrow indicates the deletion of a Q residue arising in the 6th exon, during the course of the splicing process in C57BL/6J and SEG/Pas mouse strains.

Similarly, *Csn2 *transcripts encoding β-casein were isolated from the mammary gland of SEG/Pas and C57BL/6J mice, reverse-transcribed to cDNA which were amplified and sequenced. Alignment of C57BL/6J and SEG/Pas *Csn2 *cDNA sequences (GenBank:NM_009972-1 and GenBank:GU734712, respectively) revealed the existence of 3 SNPs in the 3'UTR and 7 SNPs in the coding region, six leading to amino acid substitutions in the SEG/Pas polypeptide chain (T134A, L152V, Q160E, L174V, L181V and L208V), all occurring in exon 7 (Figure 6). Moreover, one additional transcript, showing a deletion of the first CAG codon, encoding a glutaminyl residue (Q41) in exon 6, was identified in both strains.

### Wap gene polymorphism

**Figure 7:**
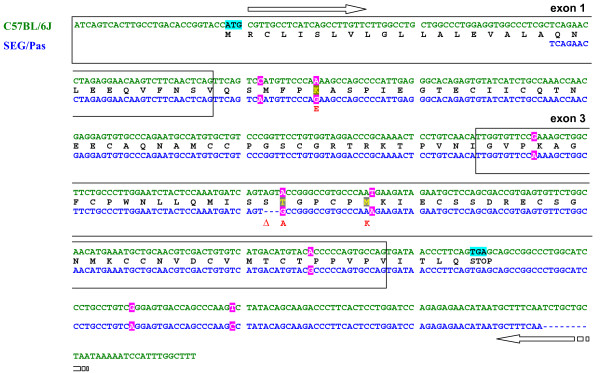
**Alignment of nucleotide and amino acid sequences of C57BL/6J and SEG/Pas *Wap***. The deduced amino acid sequence is given above according to the one-letter code. Horizontal arrows indicate the position and orientation of primers used for amplification. Pink frames show the SNP between C57BL/6J and SEG/Pas and blue frames indicate the translation-initiation and termination codon. Red amino acid residues in SEG/Pas sequence represent amino acid substitutions (K36E, T94A, M99K); Δ represents the loss of the serine in position 93.

Given the difference in electrophoresis mobility, the polymorphism of the *Wap *gene was also investigated. C57BL/6J cDNA encoding *Wap *sequenced in this work is identical to the reference mRNA sequence available in databases (GenBank:NM_011709.4). On the other hand, as shown in Figure 7, the comparison with SEG/Pas cDNA (provisory accession number GenBank:GU734713) highlights 8 SNPs in the *ca*. 450 nucleotide fragment that was sequenced. Six of them are located in the coding region while 2 are in the 3'UTR. Three SNPs are responsible for amino acid substitutions: K36E, T94A and M99K. In addition, a 3 nucleotide deletion occurring in the SEG/Pas sequence was shown to lead to the loss of a serine residue at position 93 (ΔS93).

The predicted molecular weights (monoisotopic masses) of C57BL/6J and SEG/Pas WAPs deduced from nucleotide sequences are 12,432.61 and 12,313.57 Da, respectively (Table 3). Such a weak difference (119.04 Da), due to amino acid substitution and deletion cannot account for the retarded electrophoretic mobility observed with SEG/Pas WAP in mono (SDS-PAGE) and 2D electrophoresis (Figures 4 and 3, respectively). Even though there is no evidence for post translational modifications of murine WAP in the literature, this result suggested that post translational modifications might occur in SEG/Pas WAP. To test this hypothesis, the chromatographic fractions corresponding to WAP from C57BL/6J and from SEG/Pas were collected and analysed on LTQ-Orbitrap mass spectrometer. Native masses were 12,415.60 and 12,296.54 Da, for C57BL/6J and SEG/Pas, respectively. The difference between experimental and theoretical masses for WAP from C57BL/6J and SEG/Pas is 17.01 and 17.03 Da, respectively. This mass corresponds to the formation of a pyroglutamic residue at the N-terminal position. In addition, the mass difference recorded between SEG/Pas and C57BL/6J WAPs (119.06 Da) is identical to the difference (119.04 Da) between predicted molecular weights. Therefore, no evidence for post-translational modifications of the WAP from SEG/Pas was detected.

**Table 3:** Monoisotopic mass of WAP from C57BL/6J and SEG/Pas mouse strains, obtained by tandem mass spectrometry (Orbitrap).

	Theoretical mass of WAP Monoisotopic (Da)	Experimental mass of WAP monoisotopic (Da)	Delta (Da)
**C57BL/6J**	12432.61	12415.60	-17.01
**SEG/Pas**	12313.57	12296.54	-17.03

## Discussion

Several technical approaches, including gel electrophoresis, RP-HPLC, mass spectrometry, cDNA cloning and sequencing, allowed to get a detailed description of the milk protein fraction across two mouse strains belonging to the *Mus spretus *or *Mus musculus *species. In this manner, we first observed that the protein content of *Mus spretus *milk is by far very low compared with that of C57BL/6J. In addition, we identified several polymorphisms differentiating these two species, as well as so far undescribed casein splice variants.

### The protein content varies across mouse species

Piletz and Ganschow [12], in a comprehensive study, have already reported that the milk protein concentration in fifty inbred strains of mice belonging to the *Mus musculus *species ranges between 97 g/L (in the C3H/HeJ strain) and 213.6 g/L (in the YEBT/Ha strain). Such a strain effect, affecting more generally the concentration of all milk components, was also observed across five *Mus musculus *strains [13], however to a less exent (10% of the mean milk concentration). More recently, Riley *et al. *showed that the protein concentrations in QS5i and CBA milk are 87.6 ± 7.7 and 91.6 ± 8.9 g/L, respectively [14]. Here, we show that the protein concentration in the milk of SEG/Pas mice was four folds lower compared with C57BL/6J mice and therefore similar to the protein concentration previously reported in PWK/Pas *Mus m. musculus *milk (32 ± 6 g/L) [10]. Thus, two strains recently introduced in animal facilities and belonging to *Mus spretus *and *Mus m. musculus*, display a much lower milk protein concentration by comparison with the classical *Mus m. domesticus *subspecies. Therefore, the milk protein concentration greatly varies between mouse species but also within mouse species and between strains. The range of variation between mice is quite of the same order with that observed in phylogenetically distant species, since the concentration of milk proteins may account for more than 200 g/L in some lagomorph species, whereas in human milk, it does not exceed 10 g/L [15].

### In distinct mouse strains, the composition of major milk proteins varies over the lactation period

Within species only κ-casein showed evolution in electrophoresis pattern during the course of lactation, likely corresponding to changes in the level of post-translational modifications (glycosylation). During the early stages of lactation, the SEG/Pas κ-casein gave rise to an indistinct band in SDS-PAGE suggesting some heterogeneity in the glycosylation level of the protein, as reported in QSi5 and CBA *Mus musculus *strains [14]. This pattern progressively disappeared, to finally give rise to a discrete band of a lower apparent molecular weight. This is indicative of a less glycosylated form of κ-casein in the second part of lactation. By contrast, from the beginning of lactation the less glycosylated form of C57BL/6J κ-casein was highly expressed compared with the other strains. In addition, we observed that expression of ε-casein increased from the early stages of lactation to reach a stabilized level at mid lactation (data not shown), as reported by Riley *et al. *[14]. Consistent with this, Rudolph *et al *observed low levels of ε-casein mRNA between days 1 and 2, in contrast with the expression of most milk protein genes [16].

### Protein polymorphisms distinguish Mus spretus and Mus m. domesticus milk

Of the nine major proteins from mouse milk three only, namely α_s1_-casein, β-casein and WAP, showed obvious variations in electrophoretic mobility and/or chromatographic retention time between SEG/Pas and C57BL/6J, reflecting variation in charge, hydrophobicity and molecular weight. Sequencing cDNAs encoding Csn1s1, Csn2 and Wap in both mouse species revealed differential splicing patterns and SNPs in coding sequences, of which some were responsible for amino acid substitutions. Most of the splicing variants, observed with casein mRNAs, were shared by both C57BL/6J and SEG/Pas. On the other hand, SNPs inducing amino acid substitutions are the most discriminating features to distinguish SEG/Pas from C57BL/6J.

A polymorphism in the WAP encoding gene was suspected between C57BL/6J and SEG/Pas from chromatographic as well as 2D- and 1D-electrophoresis gel behaviour. Indeed, the WAP variant in SEG/Pas is remarkably slowed and ran as a smearing spot at a molecular weight higher than expected from its amino acid sequence. From the *Wap *transcripts sequences, proteins with different molecular weights and isoelectric points (pI) are predicted: MW: 12,432.61/pI: 5.00 and MW: 12,313.57/pI: 4.83 for the C57BL/6J and SEG/Pas WAPs, respectively. The 119.04 Da difference in molecular weight cannot account for the dramatic difference in mobility of the variants on SDS-PAGE gels, whereas the horizontal shift observed in 2D electrophoresis agrees with the 0.17 difference in isoelectric points. Genetic polymorphisms across mouse strains have been previously reported for the *Wap *gene. Indeed, WAP-A and WAP-B are used to ascribe C57BL/6J and YBR variants, respectively [17]. The protein variant encoded by *WAP-B* has one cysteine less and one arginine more than the WAP-A variant. Comparison of C57BL/6J and SEG/Pas *Wap *cDNA sequences revealed mutations associated with 3 amino acid substitutions (K36E, T94A and M99K; numbering of amino acid residues is that of the pre-protein of C57BL/6J) and one amino acid deletion (ΔS93). Interestingly, the deletion of S93 together with T94A and M99K substitutions provide a domain II peptide sequence which is closer to the rat WAP than to the C57BL/6J WAP [18]. Likewise, K36E substitution, located within domain I, leads to an acidic residue (E or D) that is conserved in most species, except for the *Mus musculus *strains. Elsewhere, we found that the KSPT (or ESPT) insertion in the C-terminus part of the rat protein is due to incorporation of an intron sequence at the splice site junction between exons 3 and 4. Since these mutations in the SEG/Pas WAP are not located in the four disulfide core (4-DSC) domains containing the conserved cysteine residues, it is likely that they have small effects, if any, on the three-dimensional structure of the protein. Amino acid sequence of mouse WAPs does not highlight any potential site for post-translational modifications, in contrast to pig WAP which, from molecular weight considerations, appears to be glycosylated [19]. Neither WAP-A, nor the SEG/Pas WAP stained positively with the periodic acidic-Schiff (data not shown), suggesting that they are not glycosylated. Moreover, Orbitrap mass spectrometer data confirmed the absence of post-translational modifications in WAP from both strains. Thus the shift observed in 1D and 2D electrophoresis gels between C57BL/6J and SEG/Pas seems not to result from molecular mass alterations, but rather reflects changes in protein conformation that may affect the constant SDS/protein ratio or the shape of the SDS-protein complex. Indeed, numerous studies aimed at testing the sensitivity of electrophoresis in detecting protein polymorphisms have shown that protein migration in SDS gels is often depending on their shape which in turn varies with their conformation [5,20,21]. WAP displays a lipoprotein-like structure, although the amount of lipid associated with WAP is heterogeneous [17]. Therefore, our hypothesis is that the amount of associated lipid is higher in SEG/Pas WAP than in WAP-A, thus impairing the expected migration.

The *Wap *gene was also sequenced from strains 129/SvJ [22] and GR (GenBank:MMU 38816) belonging to *Mus musculus *species. WAP from 129/SvJ is identical to the C57BL/6J WAP-A. By contrast, WAP from GR differs from WAP-A by three amino acid substitutions (L11R, P35Q and M90T), the first one being located in the signal peptide. Thus, at least three WAP variants exist within the *Mus musculus *species and one in *Mus spretus *(this work). Following the nomenclature used by Hennighausen and Sippel [23], we propose to name WAPs from GR and SEG/Pas WAP-C and D, respectively.

The frequency of SNPs in the coding region of *Wap *cDNA from SEG/Pas and C57BL/6J was estimated to 1.76%. This frequency was only 0.5% within the coding region of *Wap *from C57BL/6J and GR that belong to the same *Mus musculus *species.

A comparison of C57BL/6J nucleotide sequences published for β- and α_s1_-caseins with the C3H/HeN and FVB/N mice strains sequences, respectively, did not reveal any genetic polymorphism. By contrast, sequencing data provided here for C57BL/6J and SEG/Pas α_s1_- and β-caseins cDNA clearly show the existence of SNPs in the coding regions, leading to 5/6 amino acid substitutions, as well as in the 3'UTRs.

Comparisons of coding and non coding (3'UTR) orthologous milk protein genes in *Mus musculus *and *Mus spretus *indicate that, on average, *Mus spretus *exhibits one SNP in every 60 to 100 bp and 20 to 50 bp, respectively. We found that SNPs occur at higher frequencies in non-coding (3'UTR: 2.86%) than in coding (1.3%) sequences in *Csn1s1*, *Csn2 *and *Wap *genes. These results agree with previous data indicating that SNPs occur at higher frequencies in non coding (2.2% and 1.4% in introns and both 3' and 5'UTRs, respectively) than in coding (0.6%) regions in *Mus spretus *[5]. However, the SNP frequencies in milk protein genes reach twice the values reported at the whole genome level. Comparing C57BL/6J and SEG/Pas, others have also estimated the polymorphism rate at the whole genome level to range between 1 and 2% [1,4].

Therefore, selection pressure seems to be lower on milk protein genes than on the rest of the genome. However, highly conserved hydrophobic domains and multiple phosphorylation sites, identified both in α_s1_- and β-caseins, are less subjected to mutations, confirming functional constraints acting to conserve the overall architecture of the corresponding molecules. Likewise, despite a significant rate of polymorphism between C57BL/6J and SEG/Pas, no mutations were detected in the WAP 4-DSC domains that are essential for its structure and function.

### α_s__1_-casein molecular diversity is mainly due to post transcriptional modifications

RP-HPLC analyses were indicative of the existence of several α_s1_-casein isoforms and polymorphisms, since α_s1_-caseins from C57BL/6J and SEG/Pas milks show different retention times. In a previous study [10], we reported that the minor and the major isoforms of α_s1_-casein exhibited different chromatographic elution behaviour between milk from C57BL/6J and PWK/Pas mice. Indeed, comparison of chromatographic profiles from C57BL/6J, SEG/Pas and PWK/Pas, revealed that the two α_s1_-casein isoforms from C57BL/6J had a shorter retention time than the isoforms from PWK/Pas and SEG/Pas milks. Despite they belong to different species, α_s1_-casein isoforms from PWK/Pas and SEG/Pas show a similar chromatographic elution pattern.

Since tryptic peptide masses that allow the identification of α_s1_-casein in both fractions did not distinguish between isoforms, native masses of proteins contained in each chromatographic fraction of α_s1_-casein were measured using MALDI-TOF mass spectrometry (data not shown). We obtained several masses ranging between 31,800 and 35,000 Da which correspond to α_s1_-casein. However, it was not possible to assign the different isoforms identified from cDNA sequencing to the molecular weights obtained, within each fraction. This result suggests the existence of additional isoforms including different phosphorylation levels. Such a hypothesis is consistent with the electrophoresis (1D) patterns of α_s1_-casein chromatographic fractions which were shown to contain at least two bands, either with C57BL/6J or SEG/Pas milks (data not shown). Bands from minor fractions migrate faster than those present in the major fractions, thus suggesting that the former contains the shortest variants (278+282 aa and 269+279 aa), whereas the major fraction contains the full-length protein (298 aa) together with protein variants arising from c (292 aa) or c'(309 aa) mRNA isoforms. Moreover, the partial deletion of exon 21 in isoforms *a *(C57BL/6J) and *a' *(SEG/Pas) should strongly modify its chromatographic behaviour since the 19-aa deleted segment encodes a number of hydrophobic residues, including 4 phenylalanyl (F68, F72, F77 and F83), 1 isoleucyl (I71) and 2 alanyl (A77 and A82) residues. This large deletion is due to the usage of a cryptic splice site occurring in exon 21, 57 nucleotides downstream from an AG defining the proper end of intron 20, in frame and following a rather strong polypyrimidine tract (n = 20) although interrupted by a triplet of contiguous G. The same deletion was also reported in the α_s1_-casein from the mouse FVB/N strain (GenBank:AAH40246). Even though such an event seems to be relatively rare, a casual improper splicing using an exon cryptic splice site and leading to the loss of 132 aa residues was also reported in the equine *CSN2 *mRNA [24]. On the other hand, the loss of a CAG, induced by an error-prone junction sequence, is much more frequent. This defect in accuracy was observed both with β-casein (already mentioned in GenBank:AK021328) and for the first time in α_s1_-casein (this work). It leads casually to the loss of a glutaminyl residue (Q) promoted by the nucleotide sequence at the junction between intron 7 and exon 8 for *Csn1s1 *mRNAs and between intron 5 and exon 6 for *Csn2 *mRNAs. The mechanism by which AG defining the 3' splice site is accurately and efficiently recognized involves a 5' to 3' scanning process [25]. The first AG downstream of the branch point-polypyrimidine tract is selected preferentially. However, the occurrence of competitive AG, downstream from the proximal one, can alternatively trigger its usage. The occurrence of a tandem CAG triplet codon at an intron-exon junction would be a facilitating feature. The casual deletion of the CAG codon was first detected in casein-coding genes in goat [26], and later in ovine [27], bovine and water buffalo [28]. Such a splice-acceptor site slippage was also reported in the human α_s1_-casein [29,30]. Examples of insertion/deletion of Q are well documented and occur in all calcium-sensitive casein pre-mRNAs, as well as in a number of other proteins in mice and humans such as ABCG8 [31,32], IGF-1 receptor [33] and PAX3 [34]. More generally, alternative splicing at short-distance tandem site is widespread in many species [35].

The insertion of 33 nucleotides upstream of exon 11 in *Csn1s1 *SEG/Pas is likely due to the usage of a cryptic splice site. Since the relevant genomic sequence of *Mus spretus *is not available yet, it is difficult to sustain such an hypothesis. However, there is an imperfect polypyrimidine tract containing several purine bases in the genomic sequence of *Mus musculus*, upstream from the 3' acceptor cryptic splice site.

**Figure 8:**
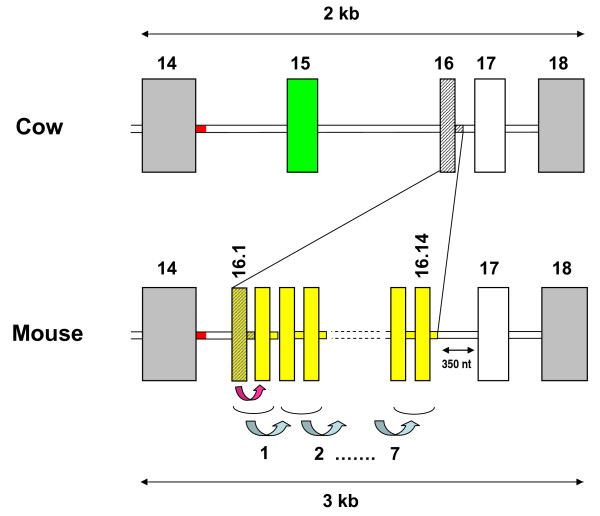
**Schematic representation of the genomic region spanning exon 14 to exon 18 of the gene encoding α**_**s1**_**-casein in cow and mouse**. Diagonally hatched block and segment in the cow genome indicate exon 16 and a part of the downstream intron which is first duplicated in the mouse genome (pink arrow). This first duplication (yellow block) is duplicated in tandem seven times in the mouse genome (blue arrow). The green block indicates exon 15 existing only in cattle, sheep and goats. The red intronic segment (30 nucleotides) appears to be conserved between several species (cattle, mice, pigs and humans). In addition in mice, there is a 350-nucleotides insertion between exon 16.14 and 17, contributing to enlarge this genome segment, as compared with cow (3 kb *vs*. 2 kb).

Additionally, several α_s1_-casein isoforms identified in C57BL/6J and SEG/Pas as splicing variants arise from "clean" skipping of exon sequences. These isoforms originated from skipping events during the processing of the primary transcripts. The phenomenon has been reported in small ruminants [26,30] and in humans [29,36]. Regarding exons 9 and 10 which are alternatively skipped, "*en bloc*" from the C57BL/6J mRNAs, this could be explained, as far as exon 9 is concerned, by weakness in the consensus sequence at the 3' acceptor splice site of intron 8. However, there is no obvious explanation for exon 10 skipping. Likewise, in SEG/Pas, two exons (16.14 and 17) were simultaneously deleted. Since genomic sequences are not available for *Mus spretus*, it is difficult to explain why this kind of event may have occurred. Exon 16.3, which is skipped in C57BL/6J, is located in a chromosomal region where major rearrangements have been reported in the mouse genome (GenBank:NC_000071). This is also the region where one can posit that exon 15 in cattle (generally in bovidae species) might have resulted from the "exonisation" of a 24 nucleotides intronic region located between exons 14 and 16, from comparison of orthologous *CSN1S1 *genes between several species. In mouse *Csn1s1*, there is a tandem repeat of 14 copies of a 18 nucleotides exon (exon 14 in Rijnkels *et al *[37] and exon 16.1 in the numbering adopted in Figure 5). Multiple alignments of genomic sequences spanning the first to the third exon copy suggest that this structure results from a first duplication event that involved exon 16 and a part of the downstream intron (Figure. 8). Later, a subsequent tandem duplication of this basic motif might have occurred seven times. This exon 16 which is present in humans [29] camel, horse, guinea-pig and pig [30,37] is also present in bovine [29]. It was shown to correspond to a short "virtual exon" occurring within intron 15 and surrounded by quite perfect consensus splice sequences, except the 5' donor splice site which is absent in the bovine genome.

About 45 different genetic variants are expressed from the 6 main bovine milk protein loci (Miranda *et al., *unpublished results) and considerable differences in allele frequencies were observed among breeds. The situation is still much more complex in less selected ruminants such as goats in which *ca*. 35 alleles have been found at *CSN1S1* and *CSN3* loci [30]. However, comparative analysis of the casein gene cluster genomic sequences across species shows that the organization and orientation of the genes is highly conserved. The conserved gene structure indicates that the molecular diversity of caseins is primarily achieved through variable species-specific use of exons (exon-skipping or differences in exon usage) and high evolutionary divergence. Caseins are the most divergent of the milk proteins with an average pairwise percent identity ranging between 44 and 55% across placental mammals [38].

By contrast to the rapid evolution of casein genes previously put forth [39], milk protein genes in general seemed to evolve more slowly than others in the bovine genome, despite selective breeding for milk production. The most conserved genes were those for proteins of the milk fat globule membrane, suggesting that the mechanism for milk-fat secretion is essential. Diversity in milk composition could not be explained by diversity of the encoded milk proteins and although gene duplication may contribute to species variation, this is not a major determinant [40]. Thus, other regulatory mechanisms must be involved. For example, on the basis of analysis of the opossum genome, Mikkelsen et al [41] concluded that most of the genomic diversity between marsupials and placental mammals comes from non-coding sequences, arising from sequence inserted by transposable elements.

Sharp et al. [42] proposed models for evolution of the WAP gene in the mammalian lineage either through exon loss from an ancient ancestor or by rapid evolution via exon shuffling, whereas a functional WAP gene has been lost in humans, cattle and goats.

The question remains however to know whether polymorphisms of milk proteins is larger between mice inbred strains than between breeds of ruminants for example?

## Conclusions

Of the nine mouse milk major proteins, only three showed variations in chromatographic retention time (α_s1_-casein, β-casein and WAP) or electrophoretic mobility (WAP) between mice species.

Considering the high frequency of SNPs between C57BL/6J and SEG/Pas, most of the other major milk proteins might be also affected by single amino acid polymorphisms (SAPs). Our hypothesis is that most of the SAPs have no consequences on the structural properties of proteins, and therefore result in "silent" polymorphism not detected by electrophoresis or chromatographic methods used.

Our results also revealed different known alternative splicing mechanisms giving rise to a large diversity of proteins of different molecular weights, isoelectric points and hydrophobicities within each mouse strains.

## Abbreviations

**WAP**: whey acidic protein; **Lf**: lactoferrin; **SA**: serum albumin; ***Csn1s1: ***α_s1_-casein gene; ***Csn2: ***β-casein gene; **aa**: amino acid; **SAP**: single amino acid polymorphisms; **UTR**: untranslated region.

## Authors' contributions

NB participated in study design, performed the RP-HPLC, 2D electrophoresis, mass spectrometry, PCR, cloning experiments and drafted the manuscript. CB helped to perform the RNA extraction and PCR experiments including primers design. ChB helped to perform 2D electrophoresis and analyzed spectra from mass spectrometry (Orbitrap). GM helped to perform RP-HPLC protocol optimization and protein identification using Peptide Map Fingerprinting. SA carried out the analysis of milk proteins over the lactation period by 1D electrophoresis. ER helped to perform cloning experiments. JJP contributed to conceive the study, provides the *M. spretus *SEG/Pas strain and helped to milk sampling as well as to draft the manuscript. SB contributed to the conception and coordination of the study and to the manuscript writing. PM contributed to conceive the study and participated in its design and coordination and helped to draft the manuscript. All authors read and approved the final manuscript.
